# Determinants of health-related quality of life: a cross-sectional investigation in physician-managed anticoagulated patients using vitamin K antagonists

**DOI:** 10.1186/s12955-020-01326-y

**Published:** 2020-03-16

**Authors:** Arianna Magon, Cristina Arrigoni, Marco Moia, Michela Mancini, Federica Dellafiore, Duilio F. Manara, Rosario Caruso

**Affiliations:** 1 Department of Biomedicine and Prevention, University of Rome Tor Vergata, Via Montpellier, 1 - 00133 Rome, Italy; 2 Department of Public Health, Experimental and Forensic Medicine, Section of Hygiene, University of Pavia, Pavia, Italy; 3 Fondazione IRCCS Ca’ Granda Ospedale Maggiore Policlinico, Angelo Bianchi Bonomi Hemophilia and Thrombosis Center and Fondazione Luigi Villa, Milan, Italy; 4 Nursing Office, ASST Melegnano e della Martesana, Melzo, Italy; 5 Health Professions Research and Development Unit, IRCCS Policlinico San Donato, San Donato Milanese, Milan, Italy; 6 School of Nursing, Vita-Salute San Raffaele University, Milan, Italy

**Keywords:** Health literacy, Oral anticoagulants, Predictors, Quality of life

## Abstract

**Background:**

Literature has paid little attention in describing the specific contribution of each modifiable and non-modifiable characteristics on health-related quality of life (HRQoL) in physician-managed anticoagulated patients using vitamin K antagonists (VKAs). To describe how patients’ treatment-specific knowledge, health literacy, treatment beliefs, clinical, and socio-demographic characteristics influence HRQoL in Italian physician-managed anticoagulated patients using VKAs.

**Methods:**

Cross-sectional multicentre study with a consecutive sampling strategy, enrolling 164 long-term anticoagulated patients. Clinical and socio-demographic characteristics were collected from electronic medical records. Valid and reliable questionnaires were used to collect patients’ treatment-specific knowledge, health literacy, beliefs about VKAs, physical and health perceptions.

**Results:**

Obtaining and understanding health information (i.e., communicative health literacy) positively predicts both adequate mental (OR_adjusted_ = 10.9; 95%CI = 1.99–19.10) and physical (OR_adjusted_ = 11.54; 95%CI = 1.99–34.45) health perceptions. Conversely, the ability to perform proper health decision making (i.e., critical health literacy) was associated with lower rates of adequate mental health perception (OR_adjusted_ = 0.13; 95%CI = 0.03–0.63). Further, age negatively predicted physical health perception (OR_adjusted_ = 0.87; 95%CI = 0.81–0.93).

**Conclusions:**

Health literacy plays an interesting role in predicting HRQoL. The relationship between critical health literacy and mental health perception could be influenced by some psychological variables, such as distress and frustration, which could be present in patients with higher levels of critical health literacy, as they could be more inclined for self-monitoring. For this reason, future research are needed to identify the most suitable patients’ profile for each OAC-management model, by longitudinally describing the predictive performance of each modifiable and non-modifiable determinant of HRQoL.

## Background

Oral anticoagulation (OAC) is a lifelong treatment that involves roughly 2 % of the western population [1]. Specifically, OAC is mainly indicated in non-valvular atrial fibrillation to prevent the incidence of ischemic stroke, as well as in other medical conditions that are associated with thromboembolic complications, such as the deep vein thrombosis [2]. An inadequate anticoagulation control is also associated with adverse events, which are severe bleeding, thromboembolic events, and mortality [3]. The most frequent adverse event is severe bleeding, showing an incidence ranging from 2 to 5% yearly, without significant differences between countries [4]. Thromboembolic complications associated with inadequate anticoagulation control show a yearly incidence of roughly 2% [5]. Mortality in anticoagulated patients is mainly associated with fatal bleeding, showing an incidence ranging from 0.5 to 1% yearly [3, 4].

Vitamin K antagonists (VKAs) have been the cornerstone of OAC for more than five decades [6]. Thus far, VKAs are the most widely used drugs for OAC for selected patients when the use of direct oral anticoagulants (DOACs) is not recommended—e.g., patients with rheumatic mitral valve disease in atrial fibrillation and/or a mechanical heart valve prosthesis [7]. The main challenges of OAC with VKAs are given by the requirements to maintain an adequate anticoagulation control, as several changes in lifestyle habits are required, such as dietary restriction, drug interactions and frequent blood monitoring [1, 8].

Literature shows two main possible strategies for OAC management: Physician-managed (i.e., usual care approach) and self-monitoring models [9]. Self-monitoring models, for their part, encompass patient self-testing and self-management: In self-testing, patients can have their test result managed by their healthcare provider; conversely, in self-management, patients can interpret their international normalized ratio (INR) result, and adjust their own dose of anticoagulant accordingly [10]. Despite self-monitoring models are a safe option for suitable patients of all ages [9], physician-managed model is still the most used approach for managing VKAs [11]. However, empirical evidence and meta-analysis synthesis showed that physician-managed model has been associated with lower levels of health-related quality of life (HRQoL) than the ones reported for self-monitoring [9, 12–14]. The interpretation of the observed differences referred to HRQoL has not found a consensus yet, supporting the hypothesis that suitable patients to self-monitoring receive more benefits when they are able to express their feeling of empowerment in managing their therapy [9].

HRQoL is an important outcome for anticoagulated patients, acknowledging that higher levels of HRQoL are associated with higher adjustment to treatment [15]. HRQoL in anticoagulated patients is generally adequate, even if there is still room for improvement [14, 16]. The individual characteristics that can be changed to achieve an improvement of HRQoL are defined as modifiable determinants, while fixed factors of an individual (e.g. genetic, age) are defined as unmodifiable determinants [17]. Literature has already shown that patients’ treatment-specific knowledge, health literacy, and treatment beliefs are the most described modifiable determinants of clinical outcomes and treatment adherence [18–20].

Treatment-specific knowledge was described as the level of patients’ awareness about risks and benefits of OAC [21] being also reported as a positive determinant of anticoagulation control [22] and being associated with better treatment adherence [19]. Furthermore, treatment-specific knowledge and adherence to OAC were described to be associated also with treatment beliefs [23]. Specifically, beliefs are defined as patients’ concerns for treatment, and their understanding of the necessity of being treated with OAC [23]. Lastly, health literacy was defined as an individual’s capacity to obtain, understand, and function the basic information and services to best manage his or her health, and engage in proper decision-making [24]. Previous research reported that anticoagulated patients with low health literacy were less adherent to OAC, exhibiting an increased risk of complication and disease-related mortality [25].

Thus far, it is not clear whether treatment-specific knowledge, health literacy, and treatment beliefs maintain their effects also in modulating the levels of HRQoL in anticoagulated patients, beyond their role in determining clinical outcomes [26]. In fact, previous research described knowledge, health literacy, and beliefs as potential modifiable determinants of HRQoL [26]. Research aimed at describing the modifiable determinants of HRQoL is pivotal for anticoagulated patients, as those determinants could be susceptible of improvement by evidence-grounded educational strategies [14, 26]. However, the role of those determinants in modulation HRQoL still remains less described than the effects of non-modifiable determinants in influencing HRQoL of anticoagulated patients. This gap is particularly important for physician-managed anticoagulated patients using VKAs, as they seem to report lower level of HRQoL than patients managed using self-monitoring [12–14]. Therefore, we aimed to identify the determinants of HRQoL in physician-managed anticoagulated patients using VKAs, highlighting the specific contribution of each non-modifiable and modifiable variable in modulating HRQoL.

## Methods

An observational study was conducted involving two anticoagulation clinics (ACs) in the north of Italy from January 2019 to July 2019. The ‘STrengthening the Reporting of OBservational studies in Epidemiology’ (STROBE) checklist was used as a guide for the study reporting (see Additional file 1).

A convenience and consecutive sampling was used to enroll adult patients with a steady coagulation profile and able to complete self-reported questionnaires through a cross-sectional data collection. A good general rule of thumb for determining desirable sample size for studies involving self-report questionnaires is to consider a minimum of 50 participants per domain to allow an adequate variation of the measurements [27]. In this study, the theoretical construct with higher number of domains was given by health literacy, as it encompasses three different sub-measurements for functional, communicative, and critical health literacy. For this reason, we calculated the desirable sample size considering the number of domains included in the Health Literacy Questionnaire (HLQ), which is described in the measurements’ paragraph [(n_domains_ = 3)*50 patients =150 patients]. According with previous research with a similar data collection approach [28], we expected a hypothetical response rate of 70%. Thus, we invited to participate in this study a total of 195 adult anticoagulated patients.

In accordance with previous research about HRQoL in real-world anticoagulated population [29], the inclusion criteria for this study were: Patients aged ≥18 years, in long-term treatment with VKAs (for at least 3 months) using physician-managed model, speaking fluently Italian. The exclusion criteria were: Patients short-term treated (< 6 months), patients who had to interrupt the treatment for a surgery in the last 3 months, with serious comorbidities (Charlson Comorbility Index [CCI] > 4), and with cognitive impairment (Six Item Screener test [SIS] < 4). Data collection was performed using paper-based case report form (CRF) in the outpatient settings of each involved hospital, and the questionnaires’ compilation required roughly 10 min.

### Measurements

Socio-demographic and clinical data (i.e., non-modifiable determinants) were detected from electronic medical records in accordance with the current guidelines, and previous research aimed at describing the anticoagulated population [7, 30]. Socio-demographics were age, sex, marital status, educational level, and occupation. Clinical variables were time in OAC, clinical indication for OAC, time in therapeutic range (TTR) computed according to Rosendaal method, anamnesis of thromboembolic or bleeding complications in the last 3 months. Modifiable determinants (i.e., knowledge, beliefs and health literacy) and HRQoL were assessed through valid and reliable self-reported questionnaires available in Italian, which were Italian Anticoagulation Knowledge Tool (I-AKT) [31], Health Literacy Questionnaire (HLQ) [32], Beliefs about Medicine Questionnaire (BMQ) [33], and Short Form survey (SF-12) [34]. These questionnaires provided a measurement of patients’ perceptions about their clinical condition and beliefs related to OAC [35].

Specifically, the Italian Anticoagulation Knowledge Tool (I-AKT) was used to measure the treatment-specific knowledge [21, 31]. It encompasses two section: the first section (*n* = 20 item) assesses the general anticoagulation knowledge for any kind of anticoagulants, while the second one (*n* = 8 item) is specific for the treatment with VKAs. The percentage rate of correct answers was computed into a final score ranging from 0 to 100%. In this study internal consistency reliability was satisfactory, as Cronbach’s α was equal to 0.738 for the overall scale.

Health Literacy Questionnaire (HLQ) is a multidimensional tool composed by 44 items, which could be categorized in three main domains in accordance with Nutbeam’s theory: functional, communicative, and critical literacy [32, 36]. These domains measure: (a) The ability of patients in being able to use basic skills of daily life functioning, such as writing and reading (i.e., functional health literacy), (b) the ability to obtain and understand health information (i.e., communicative health literacy), and (c) to perform a proper health decision making (i.e., critical health literacy). Each domain has a maximum score on 5-point Likert scale, and higher scores indicate better levels of health literacy [32]. In this study internal consistency reliability was adequate for each domain, exhibiting the following Cronbach’s α for each domain: functional health literacy = 0.773; communicative health literacy =0.945; critical health literacy = 0.900.

Beliefs about Medicines Questionnaire (BMQ) encompasses two sections related to concerns (*n* = 6 item) and necessity referred to the drug prescription (*n* = 5 item) from the patients’ perspective. The first domain assesses patients’ anxiety for VKAs, while the second domain assesses the therapeutic need as recognized by patients. The final score was computed on 5-point Likert scale for each domain [33]. In this study internal consistency reliability was adequate for each domain, showing Cronbach’s α equal to 0.799 for the domain of concerns, and 0.794 for the domain of necessity.

Finally, the short form survey (SF-12) was used to assess HRQoL. It encompasses physical component summary (PCS12), and mental component summary (MCS12) using a standardized score ranging from 0 to 100, wherein higher value indicates a more favorable health perception. According with previous research [37], it is possible to dichotomize the scores through an adequate versus an inadequate HRQoL if we consider the median split strategy [34] acknowledging that the median scores of general Italian population, clustered by different age ranges, were previously published [38]. In this study, both components showed adequate internal consistency with Cronbach’s α equal to 0.699 for PCS12 and 0.687 for MCS12.

### Ethical considerations

The institutional review board of reference of each involved centers approved the study (n. 88/INT/2018). The research methodology was performed in accordance to the ethical values of declaration of Helsinki, good clinical practice (GCP) recommendations, and European law on data privacy (GDPR 2016/679 for non-interventional studies). A written informed consent form was requested to each eligible patients before the inclusion into the study. All participants were full informed about the aims and the method of the study, as well as about the confidentiality of their responses.

### Statistical analysis

The collected variables were preliminary checked for possible missing data, outliers or errors through an analysis of frequency distribution. Qualitative variables were synthetized using frequency and percentage. Quantitative variables were initially assessed for normality using the analysis of skewness and kurtosis, followed by Shapiro-Wilk test. We employed mean ± standard deviation (SD) for the normally distributed quantitative variables. The determinants of adequate HRQoL were assessed through two logistic regression (LR) models within the framework of generalized linear models. A median split approach was used to dichotomize PCS12 and MCS12, by considering the median values previously described among Italians with an age ≥ 65 years [38]. Accordingly, PCS12 scores lower than 44.1 were considered as indicating inadequate physical health, while MCS12 scores lower than 46.4 were considered as indicating inadequate mental health. LR models were run considering (a) the correlational analysis interpretations in the first comparisons between the dichotomized PCS12, MCS12 with the collected individual-level variables (i.e., socio-demographic, clinical, knowledge, health literacy, and beliefs), and (b) the potential role of each independent variable considering previous studies [29, 39, 40]. Variables exhibiting point biserial correlation coefficient (r_pb_) with *p*-value ≤0.15 were included into the LR models as independent variables [41]. Subsequently, PCS12 and MCS12 were employed as dichotomous outcomes in the logistic regression (LR) models, after the assessment for possible collinearity between the identified independent variable by checking the strength of their bivariate associations, which should not exceed 0.45 [42]. The goodness of fit for each LR model was determined using the Hosmer-Lemeshow test and Nagelkerke’s pseudo-*R*^2^. The independent variables were entered simultaneously into the models for examining each variable’s relatively unique contribution to HRQoL. Statistical analysis was run through Statistical Package for the Social Sciences version 22 (IBM Corporation), with a two-tailed significance value set at 0.05.

## Results

### Sample

A total of 164 patients were enrolled in this study (response rate = 84.1%). The majority of patients were males (*n* = 107; 65.2%), with higher frequency of secondary educational level (*n* = 83; 50.6%), showing high rates of retired workers (*n* = 136; 82.8%), with the mean (SD) of their age equal to 74 years (SD = 10.2). The majority of patients were in treatment for more than 3 years (*n* = 145; 88.5%), and atrial fibrillation was the main clinical indication for OAC (*n* = 61; 37.2%). Only 31.7% of the enrolled patients (*n* = 52) had a TTR higher than 70% computed in the last 3 months, and clinical complications in the same period occurred in 1.2% (*n* = 2). Overall, socio-demographic and clinical data are summarized in Table 1.

**Table 1:** Socio-demographic and clinical characteristics of the sample (*n* = 164)

	*n*	*%*
Socio-demographic variables		
Sex		
Male	107	65.2
Female	57	34.8
Marital status		
Married	120	73.6
Unmarried	43	26.4
Education		
Primary school	73	44.8
Secondary school	83	50.9
Academic education	7	4.3
Occupation		
Active worker	25	15.3
Unemployed	3	1.8
Retired	136	82.9
Age		
Age Years (mean; SD)	74	10.2
Clinical variables		
TTR		
≥ 70%	52	39.4
< 70%	80	60.6
Time in treatment*		
< 6 months	3	1.8
7–12 months	3	1.8
> 12 months	12	7.4
> 36 months	145	89.0
Clinical condition for anticoagulation treatment*		
AF	61	37.6
Heart valve prosthesis (i.e., mitral or aortic valve replacement)	38	23.4
VTE	19	11.7
Stoke	2	1.3
MI	9	5.6
Others	33	20.4
Comorbidity (CCI)		
No comorbidity	3	1.9
1–2	116	71.6
3–4	43	26.5
Clinical complications (thromboembolic or haemorrhagic)		
Yes	2	1.2
No	162	98.8

*TTR* Time in Therapeutic Range, *AF* Atrial Fibrillation, *VTE* Venous Thromboembolism, *MI* Myocardial Infarction, *CCI* Charlson Comorbidity Index

Note: Missing data were reported for marital status (*N* = 1), education (*N* = 1), TTR (*N* = 32), time in treatment (*N* = 1), clinical condition for anticoagulation treatment (*N* = 2), and comorbidity (*N* = 2)

### HRQoL and self-reported variables

The self-report variables are described in Table 2. The sample exhibited a mean score of MCS12 equal to 48.3 (SD = 8.9), significantly higher than PCS12 (*P*-value< 0.001), which reported a mean score equal to 44.0 (SD = 9.6). Among the domains describing health literacy, the lowest mean scores were reported for critical health literacy (mean = 2.8; SD = 0.4; *P*-value < 0.001). Furthermore, patients’ concerns about drugs were lower than their recognition of the therapeutic need to be adherent with OAC, respectively the reported means were 3.4 (SD = 0.8) and 3.9 (SD = 0.6) (*p*-value < 0.001). The knowledge about treatment showed that the mean of correct answers of I-AKT was equal to 63.2% (SD = 12.8%).

**Table 2:** Descriptive statistics of Modifiable Determinants and HRQoL (*n* = 164)

	Mean	SD	*p*-value
Health Literacy			
Functional	3.0	0.6	< 0.001
Communicative	3.0	0.5	
Critical	2.8	0.4	
Beliefs			
Necessity	3.9	0.6	< 0.001
Concerns	3.4	0.8	
Health-related quality of Life			
PCS-12	44.0	9.6	< 0.001
MCS-12	48.3	8.9	
Knowledge			
I-AKT	63.2	12.8	*na*

*PCS12* Physical Health Composite Score of treatment related quality of life, *MCS12* Mental Health Composite Score of treatment related quality of life, *I-AKT* Italian Anticoagulation Knowledge, *na* not applicable

Table 3 describes the bivariate analysis used to identify the independent variables of the analysis for assessing the determinants of HRQoL. Adequate PCS12 (categorizing 1 = adequate PCS12 and 2 = inadequate PCS12) was more frequent when patients reported higher scores of functional health literacy (r_pb_ = −.268; *p*-value =0.001), communicative health literacy (r_pb_ = −.288; *p*-value < 0.001), critical health literacy (r_pb_ = −.255; *p*-value =0.001). Inadequate PCS12 was more frequent in patients with higher CCI (r_pb_ = .185; *p*-value =0.018), in older patients (r_pb_ = .424; *P*-value< 0.001) and among unemployed or retired patients (r_pb_ = .283; *p*-value < 0.001). Furthermore, knowledge (r_pb_ = .084; *p*-value =0.144), and concerns about drugs (r_pb_ = .117; *p*-value =0.135) reported non-significant correlations, but exhibiting *P*-values lower than 0.15.

**Table 3:** Correlations between modifiable-unmodifiable determinants and HRQoL (PCS12 & MCS12)

	PCS12 (1 = adequate; 2 = inadequate)		MCS12 (1 = adequate; 2 = inadequate)	
	r_**pb**_	***p***-value	r_**pb**_	***p***-value
CCI	.185	0.018	.010	0.896
Age	.424	< 0.001	.207	0.008
Sex (1 = male; 2 = female)	−.005	0.947	−.061	0.438
Marital Status (1 = married; 2 = unmarried)	.072	0.362	−.039	0.622
Education (1 = education equal to primary school; 2 = higher than primary school)	−.12	0.127	.002	0.977
Occupation (1 = active workers; 2 = unemployed or retired)	.283	< 0.001	.270	0.009
Time in OAC	.048	0.545	.03	0.703
TTR% (1 = adequate; 2 = inadequate)	.098	0.273	.059	0.172
I-AKT	.084	0.144	.033	0.371
HLQ_Fun	−.268	0.001	−.201	0.010
HLQ_Com	−.288	< 0.001	−.221	0.004
HLQ_Cri	−.255	0.001	−.068	0.388
BMQ_N	−.009	0.904	−.070	0.371
BMQ_C	.117	0.135	.026	0.739

*CCI* Charlson Comorbidity Index, *I-AKT* Italian anticoagulation knowledge tool, *HLQ* health literacy questionnaire, *BMQ* beliefs medicine questionnaire

Conversely, adequate MCS12 scores (categorizing 1 = adequate MCS12 and 2 = inadequate MCS12) were more frequent among patients with higher scores of functional (r_pb_ = −.201; *p*-value =0.010) and communicative health literacy (r_pb_ = −.221; *p*-value =0.004), and in younger patients (r_pb_ = .207; *p*-value =0.008), being higher in active workers (r_pb_ = .270; *p*-value =0.009).

### Determinants of HRQoL

Table 4 shows the contribution of each non-modifiable and modifiable variable in modulating HRQoL. The odds of adequate PCS12 decreased by roughly 13% for each year a participant aged (OR_adjusted_ = 0.873; 95%CI = 0.816–0.935; *p*-value < 0.001). The odds of adequate PCS12 increases by more than 11 times for each increased score of communicative health literacy (OR _adjusted_ = 11.545; 95%CI = 1.991–34.451; *p*-value =0.051).

**Table 4:** Determinants of an adequate PCS12 and MCS12 (*n* = 164)

	Adequate PCS-12				Adequate MCS-12			
	OR adjusted	95%CI		*p*-value	OR adjusted	95%CI		*p*-value
Predictors								
Comorbidity Index (CCI)	0.834	0.552	1.259	0.255	1.132	0.763	1.679	0.538
Age	**0.873**	**0.816**	**0.935**	**< 0.001**	0.949	0.896	1.006	0.080
Education (1 = education equal to primary school; 2 = higher than primary school)	0.520	0.228	1.188	0.165	0.699	0.328	1.49	0.354
Occupation (1 = active workers; 2 = unemployed or retired)	0.807	0.448	1.453	0.181	0.729	0.414	1.283	0.273
Tot I-AKT	0.985	0.957	1.014	0.061	0.997	0.97	1.025	0.849
HLQ_Functional	0.36	0.059	2.203	0.269	0.575	0.104	3.18	0.526
HLQ_Communicative	**11.545**	**1.991**	**34.451**	**0.051**	**10.929**	**1.999**	**19.102**	**0.011**
HLQ_Critical	0.729	0.14	3.796	0.708	**0.128**	**0.026**	**0.628**	**0.011**
BMQ_Concerns	0.943	0.586	1.517	0.808	1.094	0.697	1.716	0.696
Model fit								
Test di Hosmer e Lemeshow	0.097				0.391			
Pseudo-*R*^2^ (Nagelkerke)	0.391				0.223			

The odds of adequate MCS12 increases by approximately 11 times for each increased score of communicative health literacy (OR _adjusted_ = 10.929; 95%CI = 1.999–19.102; *p*-value =0.011). Furthermore, the odds of adequate MCS12 decreases by roughly 80% for each increased score of critical health literacy (OR _adjusted_ = 0.128; 95%CI = 0.026–0.628; *p*-value = 0.011).

## Discussion

This study provided an overall description of the role of modifiable and non-modifiable determinants of HRQoL in physician-managed anticoagulated patients using VKAs. Among these, health literacy showed interesting effects in determining adequate PCS12 and MCS12. Broadly, promoting adequate levels of health literacy is a public health goal for ensuring equity of care, reduction of health costs, and better achievement of outcomes [24, 43], acknowledging that health literacy is a key indicator for evaluating the quality of care delivery [44, 45].

However, in our study, health literacy showed a paradoxical effect, as critical health literacy decreased the likelihood of achieving adequate mental health perception. In accordance with previous research, critical health literacy should predict more positive outcome, such as self-care and HRQoL [24, 46, 47]. In our study, we reported that patients with higher critical thinking abilities about their chronic treatment (higher levels of critical health literacy) reported lower levels of mental health perception. To interpret this paradoxical result, we hypothesized that the physician-managed model in managing OAC could influence the relationship between critical health literacy and mental health perception. In fact, patients with higher levels of critical health literacy are often those exhibiting higher desire to take control of their health management, including treatment [48, 49]. In other words, patients with higher critical health literacy are generally inclined for self-management, as they would like to take control of their own chronic condition [50]. This suggests, possibly explaining this result, that patients with high critical health literacy can experience more frustration and distress when they are managed using traditional models for OAC, inasmuch they are not actively involved in the therapeutic decision-making [50, 51]. Notwithstanding, it should be empirically tested whether the patients with higher critical health literacy, managed using self-monitoring models, exhibit higher levels of mental health perception.

In this study, the levels of knowledge, health literacy, and recognition of the therapeutic need to be adherent with OAC are generally low. In accordance with previous evidence, more attention should thus be paid in improving the modifiable determinants of health through educational interventions, especially identifying patients that could have benefits from self-monitoring [19, 20, 23, 52, 53]. In other words, the default use of the traditional OAC management model—as it happens in the real-world clinical practice in many countries—seems to meet poorly the requirements for optimizing patients’ HRQoL through enhancing their knowledge, health literacy, and correct beliefs: One size (traditional OAC management) cannot fit all!

Thus far, the main recognized criteria to identify suitable patients for self-monitoring models were given by (a) anamnesis of adequate anticoagulation control, (b) motivation to be engaged in managing OAC through self-monitoring, (c) adequate treatment-specific knowledge, and (d) adequate cognitive functioning [7, 53]. That said, this study contributes to further understand which criteria could be useful in practice to identify suitable patients for self-monitoring, as per the role of health literacy in determining HRQoL. Health literacy was previously presented as a public health goal, explained within an integrated framework that encompasses knowledge, motivation, and cognitive functioning as personal determinants of each health literacy level (i.e., functional, communicative, and critical) [54]. This implies that the individual’s health literacy levels are shaped as the result of the relationships between personal determinants and situational factors (e.g., healthcare and social contexts) [54]. For this reason, the assessment of health literacy as a pivotal criteria for identifying suitable patients for self-monitoring could be a strategic practical implication for the international healthcare providers’ community, due to patients with higher ability to perform proper health decision making (i.e., critical health literacy) could be easily identified as suitable for self-monitoring models. Furthermore, the tools aimed at assessing health literacy levels are available in many languages, being also validated in different settings [55].

We found almost no significant relationships between HRQoL and sociodemographic and clinical characteristics, while previous evidence showed that worsen HRQoL was associated with being young, female, having lower education, and with higher comorbidity, being in short-term treatment (lower than 1 year), and with previous haemorrhagic complications [10, 12]. In our study, the age was the only sociodemographic determinant of physical health perception. This result is consistent with previous research [16, 56]. Hence, further research should in-depth clarify how socio-demographics and clinical variables modulate health perception in anticoagulated patients over time, using longitudinally collected data.

### Study limitations

This study has some limitations. Firstly, the adopted cross-sectional data collection using a limited sample did not provide information on the trajectory of HRQoL and its determinants over time, and possibly the associations between independent variables and outcomes were under-estimated. Secondly, the convenience sampling suggested caution in generalizing the results. Thirdly, the comparison between different OAC management models was not included in the study design, as only physician-managed anticoagulated patients were enrolled, acknowledging that self-monitoring models report a limited use in practice in the context of this study; however, future research should compare the effects of health literacy levels in modulating HRQoL between different OAC models. Furthermore, the role of the modifiable determinants of HRQoL could be context-specific, as some cultural and social characteristics could influence how they interact with HRQoL. For this reason, cross-national research will be useful to overcome this limit. Finally, variables related to stress perception were not collected in this study: To better understand the paradoxical result given by the association between high levels of critical health literacy and lower levels of perceived mental health, future investigations should also keep into account the assessment of stress perception.

## Conclusions

HRQoL in physician-managed VKAs anticoagulated patients seems to be associated with communicative and critical health literacy. Higher ability to obtain and understand health information about OAC is associated with more adequate physical and mental health perception. However, the patients’ critical thinking about their treatment (critical health literacy) was associated with less adequate mental health perception. This result suggests that the relationship between critical health literacy and mental health perception can be influenced by some psychological variables, such as distress and frustration, which could be present in patients with higher levels of critical health literacy, as they could be the ones more inclined for self-monitoring, acknowledging their desire to take control of their health. This hypothesis should be empirically tested to understand whether critical health literacy could be considered as criteria for identifying suitable patients for self-monitoring, by comparing its role in predicting HRQoL between physician-managed and self-monitoring models. Future research should identify the most suitable patients’ profile for each OAC-management model, by longitudinally describing the predictive performance of each modifiable and non-modifiable determinant of HRQoL.

## Supplementary information

**Additional file 1. **STROBE Statement—Checklist of items that should be included in reports of *cross-sectional studies*

## Data Availability

All data generated or analyzed during this study are included in this published article. The datasets used during the current study are available from the corresponding author on reasonable request.

## Abbreviations

OAC: Oral anticoagulation
VKAs: Vitamin K antagonists
DOACs: Direct oral anticoagulants
HRQoL: Health related quality of life
PCS12: Physical component summary
MCS12: Mental component summary
